# Optimization of Protoplast Preparation and Establishment of PEG-Mediated Genetic Transformation Method in *Cordyceps cicadae*

**DOI:** 10.3390/jof11030219

**Published:** 2025-03-13

**Authors:** Haikun Qi, Haihua Ruan, Tao Wu, Hongyang Zhang, Rui Dong, Yanjun Jiang

**Affiliations:** 1 Tianjin Key Laboratory of Food Science and Biotechnology, College of Biotechnology and Food Science, Tianjin University of Commerce, Tianjin 300134, China; qhaikun0223@163.com (H.Q.); zhyang@tjcu.edu.cn (H.Z.); 18822101174@163.com (R.D.); 2 School of Chemical Engineering and Technology, Hebei University of Technology, Tianjin 300130, China; yanjunjiang@hebut.edu.cn

**Keywords:** *Cordyceps cicadae*, protoplasts, optimization, genetic transformation

## Abstract

*Cordyceps cicadae* (*C. cicadae*) is an important edible medicinal fungus; however, owing to its wild growth and lack of genome annotation, construction of a stable genetic transformation system in *C. cicadae* is greatly limited, impeding the extensive exploitation of *C. cicadae* in industry. Here, we successfully established an efficient plasmid transformation method within protoplasts of *C. cicadae* by PEG mediation using pCas9-EGFP as a marker plasmid. In order to overcome low transformation efficiency and acquire sufficient protoplasts for transformation, the influence of enzyme species, enzymatic hydrolysis time, enzymatic hydrolysis temperature, and fungal age on protoplast preparation were analyzed sequentially, and the optimal conditions for protoplast preparation were determined as follows: 2-day-old *C. cicadae* mycelia with 1.5% lywallzyme hydrolysis at 34 °C for 5 h. Our results indicate that no less than 5.1 × 10^7^ CFU/mL protoplasts could be acquired. Additionally, five osmotic pressure stabilizers including potassium chloride (KCl), sodium chloride (NaCl), glucose, mannitol, and sucrose were employed to enhance the regeneration of protoplasts, among which sucrose exhibited the highest regeneration rate of 10.43%. The transformation efficiency of plasmid was 37.3 CFU/µg DNA. On this basis, a genetic transformation method was successfully constructed, laying the foundation for further gene editing and metabolic engineering of *C. cicadae*.

## 1. Introduction

*Cordyceps cicadae* (*C. cicadae*), also known as *Golden Cicada Flower*, *Hu Cicada*, *Big Cordyceps*, and *Cicada Antler* [1], is a traditional Chinese medicinal fungus with significant value [2]. It belongs to insect-derived medicinal fungi and forms basidiomata after infecting and parasitizing cicada larvae in the soil. *C. cicadae* is abundant with bioactive compounds such as adenosine, ergosterol, cordycepic acid, polysaccharides, and peptides [3,4]. These compounds possess diverse pharmacological properties, including immunomodulatory [5], antitumor [6], anti-fatigue, renoprotective, and antioxidative effects [7,8]. Despite its medicinal potential, the complex genome structure and lack of comprehensive gene annotation have hindered the development of stable genetic manipulation methods for *C. cicadae*, thereby limiting the deeper exploration and utilization of its resources. Consequently, it is essential to establish an efficient genetic transformation method for *C. cicadae*.

*C. cicadae* is a species of filamentous fungi. Currently, several genetic transformation methods have been widely utilized in filamentous fungi, including polyethylene glycol (PEG)-mediated protoplast transformation, Agrobacterium-mediated transformation, electroporation, and restriction enzyme-mediated integration. Among these, Agrobacterium-mediated transformation is stable and effective, but its protocols are more complex and time-consuming and may also cause chromosome rearrangement [9,10]. Electroporation requires accurate control of the transformation conditions and may not be suitable for each fungus [11]; restriction enzyme-mediated integration needs precise control of enzyme type and amount, easily resulting in non-tagged mutations [12,13]. Compared with these three methods, PEG-mediated transformation is particularly advantageous due to its low equipment requirements, straightforward protocols, high transformation efficiency, and stability of resultant transformants [14]. This method has been successfully implemented in various filamentous fungi, including *Aspergillus niger* [15], *Cordyceps militaris* [16,17], and *Beauveria bassiana* [18,19].

The cell wall of filamentous fungi is mainly composed of glucan and chitin. These substances form thick, strong regions that block the entry of exogenous genetic materials [20,21]. Hence, preparation of high-quality protoplasts is a key step in the PEG-mediated transformation method [22]. It was reported that the quality of protoplasts was influenced by the growth stage of the mycelia and the degree of cell wall removal; factors such as enzymatic hydrolysis time, enzymatic hydrolysis temperature, fungal age, and osmotic stabilizers have been systematically analyzed in previous studies of protoplast preparation [23,24]. Although basic operation protocols for PEG-mediated transformation have been reported, they are not suitable for *C. cicadae* because the cell wall components are highly variable among different strains. The optimization of transformation conditions must be based on the specific characteristics of the target fungus [25].

In this study, considering the unique cell wall composition and absence of a stable genetic transformation method for wild *C. cicadae*, we systematically optimized the conditions for protoplast preparation. Furthermore, the PEG-mediated transformation method was validated using the pCas9-EGFP plasmid as a model, aiming to establish a robust foundation for the molecular biology research of *C. cicadae*.

## 2. Materials and Methods

### 2.1. C. cicadae Strain and Main Reagents

The *C. cicadae* strain used in this study was isolated from wild *C. cicadae* collected in the Anji region of Zhejiang Province, China, and preserved in our laboratory. The strain was activated and cultured on potato dextrose agar (PDA) at 26 °C for 4 d prior to use.

The main reagents included lywallzyme (specific activity ≥ 200 U/mg, Guangzhou Institute of Microbiology, Guangzhou, China), driselase (Sigma, St. Louis, MO, USA), lyticase (specific activity ≥ 200 U/mg, Solarbio, Beijing, China), cellulase (MedChem Express, Monmouth Junction, NJ, USA), and mannitol (Solarbio, Beijing, China).

### 2.2. Preparation of Protoplasts from C. cicadae

Mycelia were harvested from PDA cultures and disrupted using a mortar and pestle. The fragmented mycelia were aseptically inoculated into 100 mL of the fermentation medium. Subsequently, the inoculated medium was incubated in an orbital shaker at 26 °C with a rotational speed of 140 rpm for 24 h. After incubation, the mycelia were collected by centrifugation at 5000 rpm for 20 min. The collected mycelia were then rinsed thoroughly with sterile water and subjected to a second centrifugation under identical conditions.

Approximately 0.01 g of mycelia were carefully mixed with 1 mL of the enzymatic lysis solution. The mixture was incubated in a shaker at 80 rpm for a predetermined duration to facilitate cell wall degradation. Following the enzymatic lysis process, the suspension was filtered through double-layer filter paper to eliminate any undigested mycelial fragments. Subsequently, the filtrate was centrifuged at 4 °C with 3000 rpm for 10 min. The supernatant was discarded, and the pellet was reconstituted with 0.8 M mannitol solution (pH 4.5).

Protoplasts were enumerated under a microscope at 400× magnification using a hemocytometer (Figure S1). Triplicate counts were performed for accuracy. Protoplast yield was calculated using the following formula [26,27,28,29]:

P = C × 4 × 10^6^ × N (1)

where P represents protoplast yield, CFU/mL; C represents the number of protoplasts per small square on the blood counting chamber, CFU/mL; and N represents the dilution ratio.

### 2.3. Optimization of Protoplast Preparation from C. cicadae

#### 2.3.1. Influence of Enzymatic Systems on Protoplast Preparation

Different types of enzymes and enzyme combinations were tested, including 1.5% (*w*/*v*) lywallzyme, 1.5% lyticase, 1.5% driselase, 1.5% cellulase, and a combination of 0.75% lywallzyme, and 0.75% driselase. The protocol was as described in Section 2.2.

#### 2.3.2. Influence of Enzymatic Hydrolysis Temperature on Protoplast Preparation

Mycelia of *C. cicadae* were tested at lysis temperatures of 32, 34, 36, 38, and 40 °C, respectively. Other procedures followed Section 2.2.

#### 2.3.3. Influence of Enzymatic Hydrolysis Time on Protoplast Preparation

The mycelia of *C. cicadae* were lysed for 1, 2, 3, 4, and 5 h. Other procedures followed Section 2.2.

#### 2.3.4. Influence of Fungal Age on Protoplast Preparation

Protoplast preparation was performed using *C. cicadae* mycelia cultured for 1, 2, 3, 4, and 5 d in a shaker. Other procedures followed Section 2.2.

### 2.4. Orthogonal Tests for Protoplast Preparation from C. cicadae

Based on the single-factor experiments, an orthogonal experimental design using the L_9_ (3^3^) table (Table 1) was employed to optimize protoplast preparation conditions. The three factors analyzed were enzymatic hydrolysis time, enzymatic hydrolysis temperature, and fungal age, with protoplast yield as the evaluation index.

### 2.5. Screening of Regeneration Media for C. cicadae Protoplasts

PDA media with different concentrations of osmotic stabilizers (KCl, NaCl, mannitol, glucose, sucrose) were used as the Regeneration Medium (RM). The protoplast suspension was adjusted to 2 × 10^4^ CFU/mL with mannitol solution, and 50 µL of diluted protoplast suspension was directly coated onto RM plates. All the inoculated plates were incubated at 26 °C for 7 d. The protoplast regeneration rate was calculated based on the number of colonies formed, using the following formula:

R = (C_1_ − C_2_)/C_3_ × 100 (2)

where R represents the regeneration rate %, C_1_ represents the regeneration of individual colonies from protoplasts, C_2_ represents the PDA number of colonies on the medium, and C_3_ represents the number of protoplasts.

### 2.6. PEG-Mediated Protoplast Transformation of pCas9-EGFP

#### 2.6.1. Construction of pCas9-EGFP Plasmid

**Figure 1:**
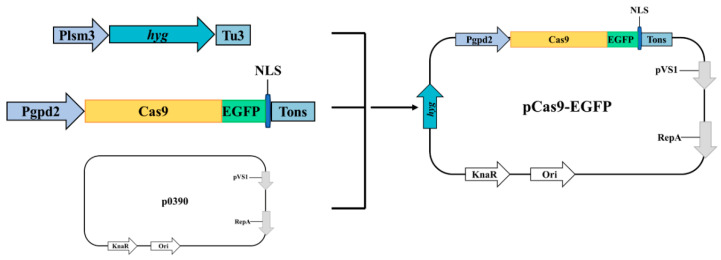
Mapping of pCas9-EGFP plasmid.

All primer sequences used in this study are listed in Table S1 of the Supplemental Material. All PCR products were amplified using PrimeSTAR GXL DNA Polymerase (TaKaRa, Kusatsu, Japan). The amino acid sequence of the Cas9 protein from *Streptococcus pyogenes* was codon-optimized for *C. cicadae*. To visualize Cas9 expression, an enhanced green fluorescent protein (EGFP) was fused to the Cas9 protein, along with a high-efficiency nuclear localization signal (NLS) to target the fusion protein to the nucleus (Figure 1, Table S2).

The initial construction utilized p390-blpR-cmcas9-gfp as the template [30]. Primers p0390R1/Pgpd2F were used to amplify the p0390-Cas9 fragment. The *Plsm3* promoter and *Tu3* terminator were amplified using primers Plsm3-F/R and Tu3-F/R, respectively, and the hygromycin resistance gene (*hyg*) was amplified from pAN7-1 using primers HYG-F/R.

Fragments were assembled using overlap extension PCR to create the *hyg* expression. The p0390-Cas9 and *hyg* fragments were subsequently incorporated into the p390 vector to construct the pCas9-EGFP plasmid (Figure 1, Table S2).

#### 2.6.2. Screening of Hygromycin B and Geneticin (G418) Concentrations

Hygromycin B and G418 are commonly used as antibiotic screening agents in fungal genetic transformation. The sensitivity of the *C. cicadae* strain to these antibiotics was evaluated on PDA medium supplemented with various concentrations of hygromycin B (0, 50, 80, 100, and 150 µg/mL) and G418 (0, 100, 200, 300, and 400 µg/mL). Control plates without antibiotics served as the negative control. Plates were incubated at 26 °C for 7 d, and growth condition was assessed.

#### 2.6.3. PEG-Mediated Protoplast Transformation of pCas9-EGFP Plasmid

Referring to Lim’s method with some modifications [31], the specific procedure for transformation was as follows. For each 1.5 mL tube containing 100 µL protoplasts, 3 µg pCas9-EGFP and 50 µL of 25% PEG4000 buffer (PEG4000 25 g, Tris-HCl 12.11 g, mannitol 14.57 g, CaCl_2_ 1 g, 100 mL) were added. The mixture was then gently mixed and incubated in an ice bath for 30 min. Subsequently, 0.5 mL of 25% PEG4000 was added to the tube. The mixture was gently mixed again and incubated at 30 °C for 20 min. Finally, the protoplasts were resuspended in 0.8 M mannitol solution, evenly spread on PDA plates containing hygromycin B, and incubated for 7 d at 26 °C.

#### 2.6.4. Microscopic Observation of pCas9-EGFP Plasmid Expression

To confirm the successful transformation of the pCas9-EGFP plasmid into *C. cicadae*, the expression of the Cas9-EGFP fusion protein was observed using a laser scanning confocal microscope (CLSM) and visualized with a Leica DMR microscope. The excitation and emission wavelengths for EGFP were 488 nm and 505–530 nm, respectively. Fluorescence indicated successful expression of the fusion protein.

Each experiment was repeated three times, with three slides examined per repetition. Five fields of view were observed per slide to ensure robust evaluation.

### 2.7. PEG-Mediated Protoplast Transformation of the G418 Gene Expression Cassette

#### 2.7.1. Construction of the G418 Gene Expression Cassette

The G418 gene fragment was synthesized in vitro. The 5′ and 3′ flanking fragments of the *URA3* gene were amplified from *C. cicadae* genomic DNA using specific primers (Table S1, Figure S2). The G418 gene fragment and the flanking fragments were seamlessly joined via cloning to construct a complete G418 gene expression cassette (Figure S2).

#### 2.7.2. Validation of the G418 Gene Expression Cassette

The *C. cicadae* protoplasts and G418 expression cassette were first mixed and then transformed according to the specific transformation method mentioned in Section 2.6.3. After that, the transformed protoplasts were coated on PDA plates containing G418 and cultured at 26 °C for 7 d. The growing mycelia were selected and transferred three times on PDA plates containing G418. Then the genomic DNA of transformants was extracted, and the primers of the G418 expression cassette were used for PCR (Table S1). The resulting PCR products were verified by agarose gel electrophoresis.

### 2.8. Statistical Analysis

All experiments were performed in triplicate. Data were analyzed using one-way analysis of variance (ANOVA) with R software (version 3.1.1, R foundation for statistical computing, Vienna, Austria). GraphPad Prism 9.5 was used for generating figures.

## 3. Results

### 3.1. Influence of Different Factors on Protoplast Preparation from C. cicadae

#### 3.1.1. Influence of Enzymes on the Preparation of Protoplasts from *C. cicadae*

**Figure 2:**
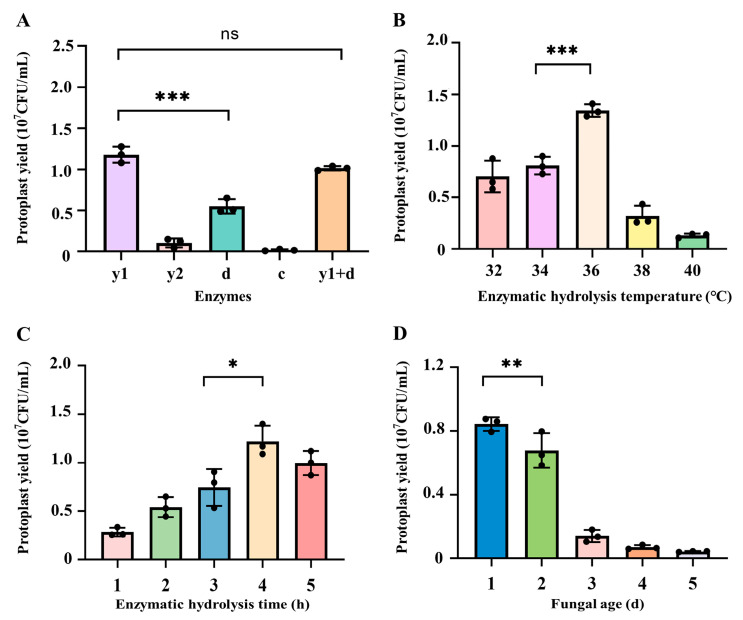
Influence of different factors on the preparation of protoplasts from *C. cicadae*. (**A**) Types of enzymes: y1: 1.5% lywallzyme, y2: 1.5% lyticase, d: 1.5% driselase, c: 1.5% cellulase, y1 + d: 0.75% lywallzyme and 0.75% driselase; (**B**) enzymatic hydrolysis temperature; (**C**) enzymatic hydrolysis time; (**D**) fungal age. Values are presented as mean ± SD (n = 3). Significance analysis between two different groups: * *p* < 0.05, ** *p* < 0.01, and *** *p* < 0.001, ns indicates no significance.

During the preparation of fungal protoplasts, based on the difference of the cell wall structure in different fungal species or even the same species distributed in different regions, various enzymes were employed for the preparation of protoplasts by enzymatic lysis [32,33]. Comprehensively summarized previous studies [34,35], the mycelia of *C. cicadae* were subjected to enzymatic lysis using different combination of enzymes, including 1.5% lywallzyme, 1.5% driselase, 1.5% lyticase, 1.5% cellulase, and 0.75% lywallzyme combined with 0.75% driselase. As illustrated in Figure 2A, the yield of protoplasts prepared after wall-broken using 1.5% lywallzyme could reach 1.18 × 10^7^ CFU/mL, which was significantly higher than that of driselase, lyticase, and cellulase under the same concentration (*p* < 0.001). Notably, the combination of 0.75% lywallzyme and 0.75% driselase produced a yield of 1.01 × 10^7^ CFU/mL, which was not statistically different from the yield achieved with 1.5% lywallzyme (*p* > 0.05). Therefore, lywallzyme was selected as the preferred enzyme for the preparation of protoplasts from *C. cicadae*.

#### 3.1.2. Influence of Enzymatic Hydrolysis Temperature on the Preparation of Protoplasts from *C. cicadae*

Temperature is one of the main factors affecting enzymatic activity, in order to determine the optimum temperature for the preparation of protoplasts from *C. cicadae*, the enzymatic hydrolysis temperature was explored in this study. As shown in Figure 2B, in the range of 32–36 °C, the yield of *C. cicadae* protoplasts gradually increased with the increase of enzymatic hydrolysis temperature. When the enzymatic hydrolysis temperature was 36 °C, the yield of *C. cicadae* protoplasts was the highest, reaching 1.34 × 10^7^ CFU/mL, which was significantly higher than that of protoplasts under the conditions of 32 °C (7.04 × 10^6^ CFU/mL) and 34 °C (8.1 × 10^6^ CFU/mL) (*p* < 0.001). When the enzymatic hydrolysis temperature exceeded 36 °C, protoplast production decreased with the continued increase in temperature, 3.2 × 10^6^ CFU/mL at 38 °C and 1.32 × 10^6^ CFU/mL at 40 °C respectively. Based on the observed trend, 34, 36, and 38 °C were selected for orthogonal optimization to further determine the optimal temperature.

#### 3.1.3. Influence of Enzymatic Hydrolysis Time on the Preparation of Protoplasts from *C. cicadae*

Insufficient enzymatic hydrolysis time leads to incomplete cell wall enzymatic lysis and a reduction in the quantity of protoplasts. Conversely, excessively long enzymatic hydrolysis time triggers the dissolution of protoplasts, resulting in a decreased protoplast regeneration rate [36]. Our results indicated that the yield of protoplasts increased rapidly with the extension of enzymatic hydrolysis time, reaching the highest yield of 1.22 × 10^7^ CFU/mL protoplasts at the enzymatic hydrolysis time of 4 h (Figure 2C). However, if the enzymatic hydrolysis time was more than 4 h, the protoplast yield began to decline, only produced 9.97 × 10^6^ CFU/mL protoplasts under 5 h enzymatic hydrolysis, likely due to protoplast degradation. Based on these findings, enzymatic hydrolysis time of 3, 4, and 5 h were selected for subsequent orthogonal optimization.

#### 3.1.4. Influence of Fungal Age on the Preparation of Protoplasts from *C. cicadae*

The culture time and status of mycelia are crucial factors influencing the efficiency of fungal protoplast preparation [37]. As shown in Figure 2D, the highest protoplast yield, 8.43 × 10^6^ CFU/mL, was achieved when the mycelia were cultured for 1 d, which was significantly higher than the yields from mycelia cultured for 2 d (6.78 × 10^6^ CFU/mL, *p* < 0.01) and 3 d (1.4 × 10^6^ CFU/mL, *p* < 0.001). A sharp decline in protoplast yield was observed with extended culture time, and almost no protoplasts were obtained under the cultivation of mycelia for 5 d. Consequently, mycelial culture durations of 1, 2, and 3 d were selected for the subsequent orthogonal optimization to determine the optimal conditions.

### 3.2. Optimization of Protoplast Preparation from C. cicadae by Orthogonal Tests

In order to further optimize the conditions for protoplast preparation, the orthogonal tests were conducted considering three factors at three levels, including fungal age, enzymatic hydrolysis time, and enzymatic hydrolysis temperature (Table 1). The results are presented in Table 2.

Based on the data demonstrated in Table 2, the order of influence on the yield of *C. cicadae* protoplasts was determined to be fungal age > enzymatic hydrolysis temperature > enzymatic hydrolysis time. The optimal conditions, as indicated by the highest k-value, were A_2_B_3_C_1_, corresponding to the fungal age of 2 d, enzymatic hydrolysis time of 5 h, and enzymatic hydrolysis temperature at 34 °C. Under these conditions, the protoplast yield reached 5.1 × 10^7^ CFU/mL.

### 3.3. Influence of Regeneration Medium on the Regeneration of C. cicadae Protoplasts

In the context of fungal protoplast studies, osmotic stabilizers play a pivotal role in maintaining cellular homeostasis by regulating osmotic pressure gradients, thereby preventing membrane rupture or structural collapse. In this study, five commonly employed osmotic stabilizers were systematically evaluated to optimize the regeneration medium for *C. cicadae* protoplasts, with the aim of enhancing protoplast viability and regeneration efficiency.

**Figure 3:**
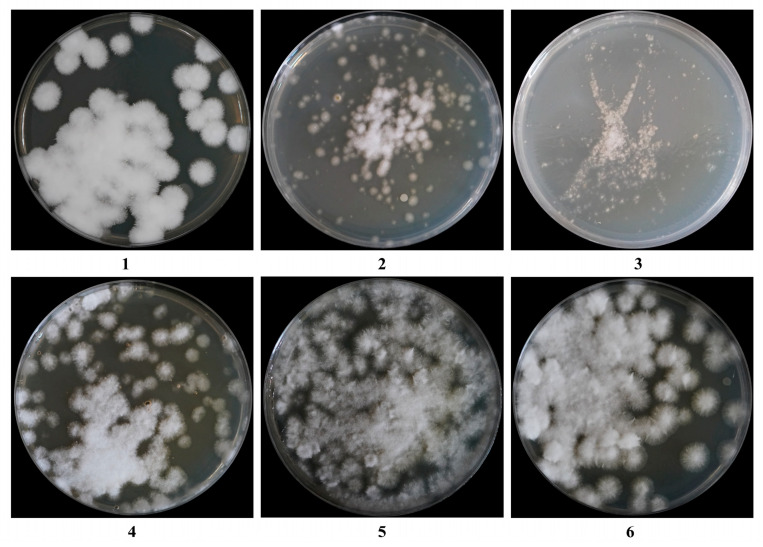
Effects of different regeneration media on protoplasts regeneration: (**1**): PDA, (**2**): PDA + KCl, (**3**): PDA + NaCl, (**4**): PDA + glucose, (**5**): PDA + sucrose, and (**6**): PDA + mannitol.

As shown in Table 3 and Figure 3, the regeneration medium contained sucrose and yielded the highest regeneration rate of 10.43%, which was significantly greater than those observed with mannitol, glucose, and the inorganic salts NaCl and KCl (*p* < 0.05, *p* < 0.001). Therefore, sucrose was selected as the osmotic stabilizer for the regeneration medium.

### 3.4. Application of PEG-Mediated Protoplasts Transformation Method in the C. cicadae

#### 3.4.1. *C. cicadae* Sensitivity Tests

**Figure 4:**
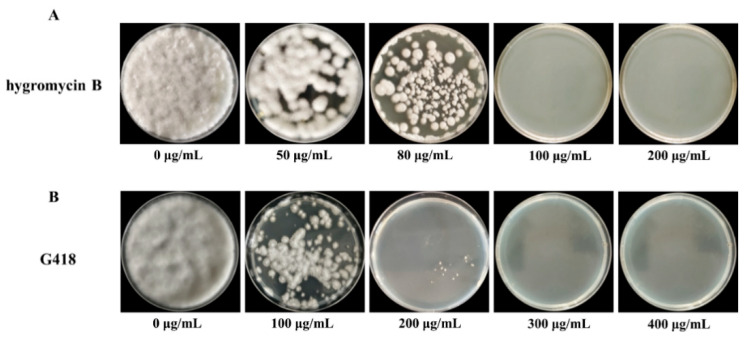
Sensitivity of wild *C. cicadae* in the PDA medium of hygromycin B (**A**) and G418 (**B**) cultured at 26 °C for 7 d.

In genetic transformation systems, the incorporation of selective agents is essential for the effective discrimination between transformants and non-transformants. In the present study, we systematically assessed the sensitivity of *C. cicadae* to hygromycin B, establishing it as a selectable marker for the identification of successful transformants in pEGFP-Cas9 plasmid transformation experiments. As shown in Figure 4A, the number of colonies decreased progressively with the increase of hygromycin B concentrations, ranging from 0 to 150 µg/mL. At a concentration of 100 µg/mL, no colony growth was observed. Therefore, 100 µg/mL of hygromycin B was selected as the optimal working concentration for plasmid transformants screening. To establish an effective genetic transformation system, we subsequently evaluated the sensitivity of *C. cicadae* to G418. As shown in Figure 4B, colony numbers declined with an increase in G418 concentrations from 0 to 400 µg/mL. At 300 µg/mL, no colony growth was observed, establishing this concentration as the optimal working level for screening transformants in subsequent experiments.

#### 3.4.2. Evaluation of PEG-Mediated Transformation of pCas9-EGFP in Protoplasts from *C. cicadae*

**Figure 5:**
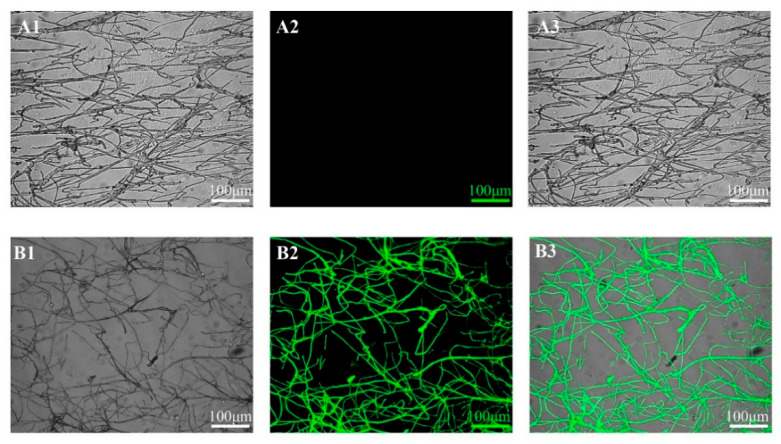
Laser scanning confocal microscope images of *C. cicadae* mycelia, among which (**A1**–**A3**) represent the control strain without plasmid and (**B1**–**B3**) represent pCas9-EGFP transformants, respectively. Bright field images (**A1**,**B1**), fluorescent images (**A2**,**B2**), merged images (**A3**,**B3**). (**A3**,**B3**) are merged images of (**A1**,**A2**) and (**B1**,**B2**), respectively. The bars = 100 µm.

The pCas9-EGFP plasmid encodes a fusion protein combining Cas9 and EGFP, allowing fluorescence intensity to indicate successful protein expression after successful plasmid transformation (Figure 1). As shown in Figure 5, transformants containing the pCas9-EGFP plasmid exhibited significantly more green fluorescence spots and enhanced fluorescence intensity compared to control mycelia. These results confirmed the successful expression of the EGFP protein, demonstrating that the pCas9-EGFP plasmid was effectively transformed into *C. cicadae*. The protoplast transformation efficiency of pCas9-EGFP was 37.3 CFU/µg DNA (Figure S3). These results validate the feasibility of the PEG-mediated genetic transformation method.

#### 3.4.3. Evaluation of PEG-Mediated Transformation of G418 Expression Cassette in Protoplasts from *C. cicadae*

**Figure 6:**
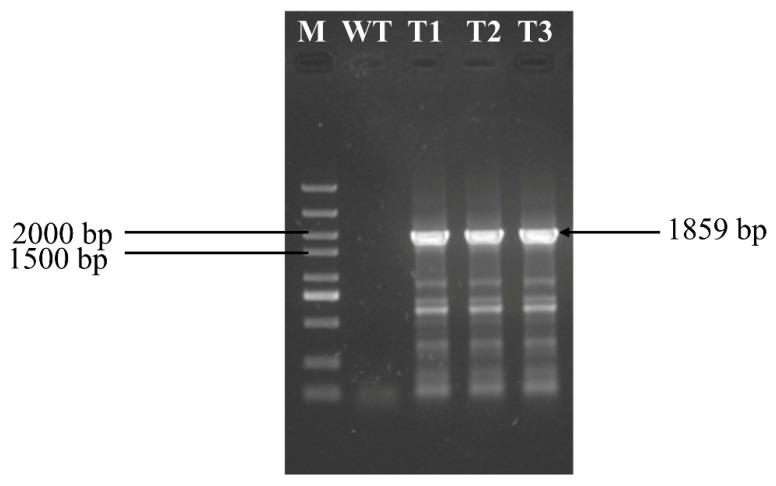
Identification of transformants (M: marker; WT: wild-type strain; T1, T2, and T3: transformants 1, 2, and 3, respectively).

To further validate the feasibility of the PEG-mediated transformation method, the G418 expression cassette was transformed into protoplasts from *C. cicadae*. Single colonies isolated on PDA medium containing G418 underwent three rounds of screening on antibiotic-containing medium and were then randomly selected for PCR verification. The G418 resistance gene was successfully amplified in the genomic DNA of the transformed *C. cicadae* (Figure 6), whereas no amplification was observed in the wild type. The protoplast transformation efficiency of G418 expression cassette was 66.67 CFU/µg DNA (Figure S3). These findings not only validate the reliability of the established transformation system but also provide a robust platform for subsequent genetic manipulation and functional genomics studies in *C. cicadae.*

## 4. Discussion

A stable genetic manipulation method is a basement for research on microbial-derived natural products. In this study, we successfully developed preparation and regeneration protocols for *C. cicadae* protoplasts and established a PEG-mediated transformation method.

For filamentous fungi, the establishment of a stable genetic transformation system relies on the successful isolation and regeneration of protoplasts [38]. A key factor in protoplast preparation is the enzyme used for cell wall lysis. The selection of a suitable, high-activity enzyme facilitates more effective mycelial wall degradation and increases protoplast yield. Our results demonstrated that enzymatic lysis using lywallzyme from the Guangzhou Institute of Microbiology and driselase from Sigma yielded high-quality protoplasts with complete mycelial cleavage. This efficiency may be attributed to the suitability between the cell wall composition of *C. cicadae* and the enzyme composition. Lywallzyme, derived from *Trichoderma longibrachiatum*, contains protease, chitinase, and cellulase activities, making it highly effective for basidiomycetes and ascomycetes. Driselase, a composite enzyme containing laminarinase, xylanase, and cellulase, has been widely used for protoplast preparation in phytopathogenic fungi [39]. Our study showed that 1.5% lywallzyme alone could reach the highest yield of 1.18 × 10^7^ CFU/mL protoplasts in 4 h enzymatic hydrolysis; thus, it was selected in subsequent experiments. Importantly, the optimization of lysis conditions was identified as a critical factor influencing the efficiency of protoplast preparation. Comprehensive analysis revealed that three key parameters—fungal age, enzymatic hydrolysis time, and enzymatic hydrolysis temperature exerted significant impacts on protoplast yield. The fungal age impacts cell wall structure, mycelial metabolism, and vigor. Insufficient fungal age results in thin, fragile cell walls, reducing protoplast yield and regeneration rate, while excessive age thickens the cell wall, hindering lysis [32,37]. Similarly, enzymatic hydrolysis time affects mycelial lysis. Incomplete lysis limits protoplast yield, whereas prolonged lysis leads to protoplast rupture and insufficient residual cell wall material for regeneration [40]. Enzymatic hydrolysis temperature influences enzyme activity, and optimal temperature maximizes enzyme efficiency. Through orthogonal tests, the optimal conditions were identified as 1.5% lywallzyme at 34 °C for 5 h, using 2-day-old mycelia, yielding 5.1 × 10^7^ CFU/mL of protoplasts (Table 2). Compared with previous reports [23,28,37,41,42], our protoplast production was at a relatively higher level. Subsequently, the screening of protoplast regeneration medium was conducted, and sucrose-containing medium was identified as the optimal regeneration medium, achieving the highest regeneration rate (10.43%, Table 3). This observation demonstrates remarkable consistency with the findings reported by Wu and Vellaisamy [24,43], which highlighted the protective role of sugars in maintaining protoplast integrity and promoting cell wall regeneration [44]. Different fungi may require different stabilizers, for example, sorbitol supports protoplast regeneration in *Cordyceps militaris* [23], mannitol in *Cordyceps sinensis* [45], and KCl in *A. niger* [25].

For effective genetic transformation, the selection of appropriate screening markers and precise determination of inhibitory concentrations are both critical parameters [46]. Hygromycin B is widely used in filamentous fungi, with effective concentrations ranging from 50 to 400 µg/mL [47,48]. In this study, 100 µg/mL was identified as the minimum inhibitory concentration for *C. cicadae* (Figure 4). PEG-mediated transformation method involves three key steps: protoplast generation, plasmid uptake, and growth of transformants on selective medium [49]. Based on the previous reports of PEG concentration (200–400 g·L^−1^ PEG 4000) used in transformation [50,51], 250 g/L PEG-mediated transformation at 30 °C for 20 min was constructed and we successfully introduced the pCas9-EGFP plasmid into *C. cicadae* protoplasts. The robust fluorescence signal observed confirmed the successful transformation (Figure 5). Since different fungi generally have unique and complex cell wall structures, it is not clear whether our method can be applied to other fungal species. Other genetic transformation methods will also need to be studied and compared.

## 5. Conclusions

In conclusion, the optimal conditions for preparing protoplasts from *C. cicadae* were determined: 2-day-old mycelia with 1.5% lywallzyme hydrolysis at 34 °C for 5 h, achieving a yield of 5.1 × 10^7^ CFU/mL. The highest regeneration rate (10.43%) was obtained using sucrose-containing regeneration medium. The successful transformation of the pCas9-EGFP plasmid and G418 expression cassette demonstrates the feasibility of the PEG-mediated method, paving the way for further molecular genetic studies in *C. cicadae*.

## Figures and Tables

**Table 1:** Factors and levels of orthogonal optimization.

Factors	Symbol	Levels		
		1	2	3
Fungal Age (d)	A	1	2	3
Enzymatic Hydrolysis Time (h)	B	3	4	5
Enzymatic Hydrolysis Temperature (°C)	C	34	36	38

**Table 2:** Orthogonal test results of protoplast preparation from *C. cicadae*.

Experimental Number	A Fungal Age/d	B Enzymatic Hydrolysis Time/h	C Enzymatic Hydrolysis Temperature/°C	Protoplast Yield (CFU/mL × 10^6^)
1	1	3	34	15.30
2	1	4	38	3.55
3	1	5	36	8.00
4	2	3	38	5.50
5	2	4	36	23.50
6	2	5	34	51.00
7	3	3	36	1.00
8	3	4	34	1.40
9	3	5	38	0.25
k1	8.95	7.26	22.56	
k2	26.67	9.48	3.10	
k3	0.88	19.75	10.80	
R	25.78	12.49	19.46	

k represents the average of the experimental results for a factor at a given level. R denotes the extreme variance.

**Table 3:** Effects of different regeneration media on the regeneration rate of protoplasts.

Regeneration Media	Protoplast Regeneration Rate (%)
PDA	control
PDA + KCl	2.87 ± 0.51 d
PDA + NaCl	1.39 ± 0.96 d
PDA + glucose	4.78 ± 0.85 c
PDA + sucrose	10.43 ± 1.18 a
PDA + mannitol	7.11 ± 1.03 b

Different letters indicate a significant difference with *p* < 0.05, as determined with one-way ANOVA.

## Data Availability

The original contributions presented in this study are included in the article/Supplementary Materials. Further inquiries can be directed to the corresponding authors.

## Supplementary Materials

The following supporting information can be downloaded at https://www.mdpi.com/article/10.3390/jof11030219/s1, Figure S1: Protoplasts from *C. cicadae*; Figure S2: Construction of G418 expression cassette; Figure S3: Colonies growth on PDA medium plates after 7 d of incubation; Table S1: Primers used in this study; Table S2: Sequences synthesized for pCas9-EGFP and G418 expression cassette.
