# Supplementary material for: Optimization of Protoplast Preparation and Establishment of PEG-Mediated Genetic Transformation Method in Cordyceps cicadae
Source: J Fungi (Basel). 2025 Mar 13;11(3):219. doi: 10.3390/jof11030219 (PMC11943176; doi:10.3390/jof11030219)
Supplement: Supplementary file 1 [file jof-11-00219-s001.zip › jof-3496613-supplementary.pdf]

Optimization of Protoplast Preparation and Establishment of PEG-mediated Genetic Transformation Method in *Cordyceps cicadae*

Table S1 Primers used in this study

Primers for pCas9-EGFP construction	Sequences (5'→3')
Plsm3-F	tgtcttttcgcgcggcgg
Plsm3-R	tatgaaattatgagcgctctgttgtg
HYG-F	atgcctgaactcaccgcg
HYG-R	ctattcctttgccctcggacg
Tu3-F	tggcaccacttcgtcttc
Tu3-R	tcaaaaaggttatcagggacgtctacg
Pgpd2-F	ggccaatttgtctattctggtagttcc
P0390-R	ctagggaattaattcggcgttaattcag

Primers for G418 expression cassette	Sequences (5'→3')
up-F	ATGGCGTCTCATCCGACTCTCAAGGC
up-R	CCGCCGCGCGAAAAGACAGCGGTCTTCGAAGATGAGGAAG
G418-F	CTCATCTTCGAAGACCGCTGTCTTTTCGCGCGGC
G418-R	GGCTTTGCCGGGAACCATTCAAAAAGGTTATCAGGGACGTCT
down-F	CCCTGATAACCTTTTTGAATGGTTCCCGGCAAAGC
down-R	GAACAGCATCTCGTCGCCTTCGC

Primers for PCR	Sequences (5'→3')
G418-F	CTCATCTTCGAAGACCGCTGTCTTTTCGCGCGGC
G418-R	GGCTTTGCCGGGAACCATTCAAAAAGGTTATCAGGGACGTCT

Table S2 Sequences synthesized for pCas9-EGFP and G418 expression cassette

1. The sequences of pCas9-EGFP:
Purple: Cas9
Orange: linker
Green: EGFP
GGCCAATTTGTCTATTCTGGTAGTTCCGCCGTGGCCAACTTGACGGTTCACTGGTTTT<br>GTGTCTTGATGACTGACAAAGAGGGCCAGAGATGAGACGACGAGCTGCCGGTTGC<br>ACCATCAGCCAACCGTTCGCAGAAATCATCAGTTGCGCTACAGTCAACATAGCGGGT<br>GGTCAAAGTTTGCGTGGGCACGGGTTGCCGCACGAGTTGGAACGTCAGTAAAGTGG<br>GGGAGGGGAAGCAACCAGAACGAGGCCAATTGAATGGGATGAAGGCGTCTGGGAG<br>GGAAGCACGAGCGTGAATGAATGGACGGTTGCGTGACTCTGGTCTCGCGATTTCGA<br>TCGAGTCAAGGAACCCGTATGGTTGCCTCTTGTCTTGGAGAGCTCGTCGAGGCCCA<br>AGCTGCGAGTATATGGTTGGTGATGCGCTGGGCATTTGCCCCTCTCTGCCCCTCCATG<br>GACGGACCTGCAGCTCTTGGGCTGGCAAGGTGTTGCGCTACGTCGAGATGCGGTTA<br>GGTAAGCTGGCATAAGCTGGCATAAGGTTGGCTGTTCTTGAGAAATCAGGCCAGCA<br>GTCTTGCACCTCTCTGGCTACAGGCTCTCCAGCGGTCAGCCACCGCCACTCCAGCG<br>CCTGTTTTTCACTGACGATGGCCTTGTTTTTTTGTTGCTCTGGCAGCTGGCAAGCTG<br>GGTCCCGGCAGCTGCCACGGCCAAGCCACCCAGGACTACAAAACCATTTGCCCACC<br>CCTCCTCTTCGCCGTGCGTCTCTTCTTTTTCTCCCCTCCATTACACCTCATTCTTCAGT<br>TCAAAGGAACAAAAGTGAGTTTTCCATCCGTCCCACACCTGGCTCCATCATCTCGAG<br>CTTCATTGCTTCTTGACCATCTACCACGCTCTACCCTCCAACAGCTTCGCTCACCCAA<br>GAGCTGCCTGCTTTTCCCCTCCTCTCCACGACCCCGCCATCCACCACTTAACCTAAA<br>GTTACCCCTCGCTAACCGCCAAACGTCCCAACAGGTTTTTCTTCTCTCACCTCACTTT<br>TCTAATCAAGAACAACTAGTATGGACAAGAAGTACAGCATCGGCCTCGACATCGGC<br>ACCAACAGCGTCGGCTGGGCCGTCATCACCGACGAGTACAAGGTCCCGAGCAAGA<br>AGTTCAAGGTCCTCGGCAACACCGACCGCCACAGCATCAAGAAGAACCTCATCGGC<br>GCCCTCCTGTTCGATTCCGGCGAGACCGCCGAGGCGACCCGGCTCAAACGCACGGC<br>CCGCCGGCGCTATACGCGGCGCAAGAACCGCATCTGCTACCTCCAAGAGATCTTCAG<br>CAACGAGATGGCCAAGGTCGACGACAGCTTCTTCCACCGCCTGGAGGAGAGCTTCC<br>TCGTCGAGGAGGACAAGAAGCACGAGCGCCACCCCATCTTCGGCAACATCGTCGAC<br>GAGGTCGCCTACCACGAGAAGTACCCCACCATCTACCACCTCCGCAAGAAGCTCGT<br>CGACAGCACCGACAAGGCCGACCTCCGCCTCATCTACCTCGCCCTCGCCCACATGAT<br>CAAGTTCCGCGGCCACTTCCTCATCGAGGGCGACCTCAACCCCGACAACAGCGACG<br>TCGACAAGCTCTTCATTCAGCTCGTGCAGACCTACAATCAGCTCTTCGAGGAGAACC<br>CCATCAACGCGAGCGGCGTCGACGCGAAGGCCATCCTCAGCGCCCGCCTCAGCAAG<br>AGCCGCCGCCTCGAAAACCTCATCGCGCAGCTCCCCGGCGAGAAGAAGAACGGCC<br>TCTTCGGCAACCTGATCGCCCTCAGCCTCGGCCTCACCCCCAACTTCAAGAGCAACT<br>TCGACCTCGCCGAGGACGCCAAGCTGCAGCTCAGCAAGGACACCTATGACGATGAC<br>CTCGACAATCTCCTCGCGCAGATCGGCGATCAGTACGCCGACCTCTTCCTCGCCGCC<br>AAGAACCTCAGCGACGCCATCCTCCTGTCCGACATCCTCCGCGTCAACACCGAGATT<br>ACCAAGGCGCCCCTCAGCGCGAGCATGATCAAGCGCTACGACGAGCACCACCAAG<br>ACCTCACCCTCCTCAAGGCCCTCGTCCGGCAGCAGCTCCCCGAGAAGTACAAGGAG<br>ATCTTCTTCGATCAGAGCAAGAACGGCTACGCCGGCTACATCGACGGCGGCGCGAG<br>CCAAGAGGAGTTCTACAAGTTCATCAAGCCCATCCTGGAGAAGATGGACGGCACCG<br>AAGAGCTCCTCGTGAAACTCAACCGCGAGGATCTCCTCCGCAAGCAGCGCACCTTC<br>GACAACGGCAGCATCCCCCATCAGATCCACCTCGGCGAGCTGCACGCCATTCTGCG<br>CCGCCAAGAGGACTTCTACCCCTTCCTCAAGGACAACCGCGAGAAGATCGAGAAG<br>ATCCTCACCTTTCGCATCCCCTACTATGTGGGCCCCCTCGCCCGCGGCAACAGCCGC<br>TTCGCCTGGATGACCCGCAAGAGCGAGGAGACCATCACCCCCTGGAACTTCGAAGA<br>AGTCGTCGACAAGGGCGCGAGCGCGCAGAGCTTCATCGAGCGCATGACCAACTTCG<br>ACAAGAACCTCCCGAACGAGAAGGTGCTCCCCAAGCATAGCCTCCTCTACGAGTAC<br>TTCACCGTCTACAACGAGCTCACCAAGGTCAAGTACGTCACCGAGGGCATGCGCAA<br>GCCCGCCTTCCTCAGCGGCGAGCAGAAGAAGGCCATCGTCGATCTCCTCTTCAAGA<br>CCAACCGCAAGGTCACCGTCAAACAGCTCAAGGAGGACTACTTCAAGAAGATCGA<br>GTGCTTCGACAGCGTCGAGATCTCCGGCGTCGAGGACCGCTTCAACGCGAGCCTCG

GCACCTACCACGATCTCCTCAAGATCATCAAGGACAAGGACTTCCTCGACAACGAG
GAGAACGAGGACATCCTGGAGGACATCGTCCTCACCCTCACCCTCTTCGAGGACCG
CGAGATGATCGAGGAGCGCCTCAAGACCTACGCCCACCTCTTCGACGACAAGGTCA
TGAAACAACTCAAGCGCCGGCGCTACACGGGCTGGGGCCGCCTCAGCCGCAAGCT
CATCAACGGCATCCGCGACAAGCAGAGCGGCAAGACCATCCTCGACTTCCTCAAGA
GCGACGGCTTCGCCAACCGCAACTTCATGCAGCTCATCCACGACGACAGCCTCACC
TTCAAGGAAGACATTCAGAAGGCCCAAGTCAGCGGCCAAGGCGACAGCCTCCACG
AGCACATCGCCAACCTCGCCGGCAGCCCCGCCATCAAGAAGGGCATCCTGCAGACC
GTCAAGGTCGTCGACGAGCTCGTCAAGGTCATGGGCCGCCACAAGCCCGAGAACAT
CGTCATCGAGATGGCCCGCGAGAATCAGACCACGCAGAAGGGTCAGAAGAACAGC
CGCGAGCGCATGAAGCGCATCGAGGAGGGCATCAAGGAGCTCGGCAGCCAAATCCT
CAAGGAGCACCCCGTCGAGAACACGCAGCTGCAGAACGAGAAGCTCTACCTCTACT
ACCTGCAGAACGGCCGCGACATGTACGTCGACCAAGAGCTCGACATCAACCGCCTC
AGCGACTACGACGTCGACCACATCGTCCCGCAGAGCTTTCTCAAGGACGACAGCAT
CGACAACAAGGTCCTCACCCGCAGCGACAAGAACCGCGGCAAGAGCGACAACGTC
CCGAGCGAAGAGGTCGTCAAGAAGATGAAGAATTACTGGCGGCAGCTCCTCAACG
CCAAGCTCATCACGCAGCGCAAGTTCGACAACCTCACCAAGGCCGAGCGCGGCGG
CCTCAGCGAGCTCGACAAGGCCGGCTTCATCAAGCGGCAGCTCGTCGAGACCCGGC
AGATCACCAAGCACGTCGCGCAGATCCTCGACAGCCGCATGAACACCAAGTACGAC
GAGAACGACAAGCTCATCCGCGAGGTCAAGGTCATCACCCTCAAGAGCAAGCTCGT
CAGCGACTTCCGCAAGGACTTTCAGTTCTACAAGGTCCGCGAGATCAACAACTACC
ACCACGCCCACGACGCCTACCTCAACGCCGTCGTCGGCACCGCCCTGATCAAGAAG
TACCCGAAACTGGAGAGCGAGTTCGTCTACGGCGACTACAAGGTCTACGACGTCCG
CAAGATGATCGCCAAGAGCGAGCAAGAGATCGGCAAGGCCACCGCCAAGTACTTCT
TCTACAGCAACATCATGAACTTTTTCAAGACCGAGATCACCCTCGCGAACGGCGAG
ATCCGGAAGCGCCCCCTCATCGAGACCAACGGTGAGACCGGCGAGATCGTCTGGGA
CAAGGGCCGCGACTTCGCCACCGTCCGCAAGGTCCTCAGCATGCCCCAAGTCAACA
TTGTCAAAAAGACCGAGGTGCAGACCGGCGGCTTCAGCAAGGAGAGCATCCTGCC
GAAGCGCAACAGCGACAAGCTGATCGCGCGCAAGAAGGACTGGGACCCCAAGAAG
TACGGCGGCTTCGACAGCCCCACCGTCGCCTACAGCGTCCTCGTCGTCGCCAAGGT
CGAGAAGGGCAAGAGCAAGAAGCTCAAGAGCGTCAAGGAGCTCCTCGGTATCACC
ATCATGGAGCGCAGCAGCTTCGAGAAGAACCCCATCGACTTCCTGGAGGCCAAGGG
CTACAAAGAGGTCAAGAAGGACCTCATCATCAAGCTCCCCAAGTACAGCCTCTTCG
AGCTGGAGAACGGCCGCAAGCGCATGCTCGCGAGCGCCGGTGAGCTGCAGAAGGG
CAACGAGCTCGCCCTCCCGAGCAAGTACGTCAACTTCCTCTACCTCGCGAGCCACT
ACGAGAAGCTCAAGGGCAGCCCCGAGGACAACGAGCAGAAGCAGCTCTTCGTCGA
GCAGCACAAGCACTACCTCGACGAGATCATCGAGCAGATCAGCGAGTTCAGCAAGC
GCGTCATCCTCGCCGACGCCAACCTCGACAAGGTGCTGAGCGCCTACAACAAGCAC
CGCGACAAGCCCATCCGCGAGCAAGCCGAGAACATCATCCACCTCTTCACCCTCAC
CAACCTCGGCGCCCCCGCCGCCTTCAAGTACTTCGACACCACCATCGACCGCAAGC
GCTACACGAGCACCAAAGAGGTCCTCGACGCCACCCTCATCCACCAAAGCATCACC
GGTCTCTACGAGACCCGCATCGACCTCAGCCAACTCGGTGGTGACGAGGGCGCCGT
CAGCAAGGGCGAGGAGCTCTTCACCGGCGTCGTCCCCATCCTCGTCGAGCTCGACG
GCGACGTCAACGGCCACAAGTTCAGCGTCAGCGGCGAGGGCGAGGGCGACGCCAC
CTACGGCAAGCTCACCCTCAAGTTCATCTGCACCACCGGCAAGCTCCCCGTCCCCTG
GCCCACCCTCGTCACCACCCTCACCTACGGCGTGCAGTGCTTCAGCCGCTACCCCGA
CCACATGAAGCAGCACGATTTCTTCAAGAGCGCCATGCCCGAGGGCTACGTCCAAG
AGCGCACCATCTTCTTCAAGGACGACGGCAACTACAAGACCCGCGCCGAGGTCAAG
TTCGAGGGCGACACCCTCGTCAACCGCATCGAGCTCAAGGGTATCGACTTCAAGGA
GGACGGCAACATCCTCGGCCACAAGCTGGAGTACAACTACAACAGCCACAACGTCT
ACATCATGGCCGACAAGCAGAAGAACGGCATCAAGGTCAACTTCAAGATCCGCCAC
AACATCGAGGACGGCAGCGTGCAGCTCGCCGACCACTATCAGCAGAACACCCCCAT
CGGCGACGGCCCCGTCCTCCTCCCCGACAACCACTACCTCAGCACGCAGAGCGCCC
TGAGCAAGGACCCCAATGAGAAGCGCGACCACATGGTCCTCCTGGAGTTCGTCACC
GCCGCCGGCATCACCCTCGGCATGGACGAGCTCTACAAGCCCGCCGCCAAGCGCGT
CAAGCTCGACTGAGGTACCACGCGTACTAGTGTTAACGCTAGCCACCACCACCACC
ACCACGTGTGAATTACAGGTGACCAGCTCGAATTTCCCCGATCGTTCAAACATTTGG

CAATAAAGTTTCTTAAGATTGAATCCTGTTGCCGGTCTTGCGATGATTATCATATAATT
TCTGTTGAATTACGTTAAGCATGTAATAATTAACATGTAATGCATGACGTTATTTATGA
GATGGGTTTTTATGATTAGAGTCCCGCAATTATACATTTAATACGCGATAGAAAACAA
AATATAGCGCGCAAACTAGGATAAATTATCGCGCGCGGTGTCATCTATGTTACTAGAT
CGGGAATTAAACTATCAGTGTTTGACAGGATATATTGGCGGGTAAACCTAAGAGAAA
AGAGCGTTTATTAGAATAACGGATATTTAAAAGGGCGTGAAAAGGTTTATCCGTTCG
TCCATTTGTATGTGCATGCCAACCACAGGGTTCCCCTCGGGATCAAAGTACTTTGATC
CAACCCCTCCGCTGCTATAGTGCAGTCGGCTTCTGACGTTCAGTGCAGCCGTCTTCT
GAAAACGACATGTCGCACAAGTCCTAAGTTACGCGACAGGCTGCCGCCCTGCCCTT
TTCCTGGCGTTTTCTTGTCGCGTGTTTTAGTCGCATAAAGTAGAATACTTGCGACTAG
AACCGGAGACATTACGCCATGAACAAGAGCGCCGCCGCTGGCCTGCTGGGCTATGC
CCGCGTCAGCACCGACGACCAGGACTTGACCAACCAACGGGCCGAACTGCACGCG
GCCGGCTGCACCAAGCTGTTTTCCGAGAAGATCACCGGCACCAGGCGCGACCGCCC
GGAGCTGGCCAGGATGCTTGACCACCTACGCCCTGGCGACGTTGTGACAGTGACCA
GGCTAGACCGCCTGGCCCGCAGCACCCGCGACCTACTGGACATTGCCGAGCGCATC
CAGGAGGCCGGCGCGGGCCTGCGTAGCCTGGCAGAGCCGTGGGCCGACACCACCA
CGCCGGCCGGCCGCATGGTGTTGACCGTGTTCGCCGGCATTGCCGAGTTCGAGCGT
TCCCTAATCATCGACCGCACCCGGAGCGGGCGCGAGGCCGCCAAGGCCCGAGGCGT
GAAGTTTGGCCCCCGCCCTACCCTCACCCCGGCACAGATCGCGCACGCCCGCGAGC
TGATCGACCAGGAAGGCCGCACCGTGAAAGAGGCGGCTGCACTGCTTGGCGTGCAT
CGCTCGACCCTGTACCGCGCACTTGAGCGCAGCGAGGAAGTGACGCCCACCGAGG
CCAGGCGGCGCGGTGCCTTCCGTGAGGACGCATTGACCGAGGCCGACGCCCTGGCG
GCCGCCGAGAATGAACGCCAAGAGGAACAAGCATGAAACCGCACCAGGACGGCCA
GGACGAACCGTTTTTCATTACCGAAGAGATCGAGGCGGAGATGATCGCGGCCGGGT
ACGTGTTCGAGCCGCCCGCGCACGTCTCAACCGTGCGGCTGCATGAAATCCTGGCC
GGTTTGTCTGATGCCAAGCTGGCGGCCTGGCCGGCCAGCTTGGCCGCTGAAGAAAC
CGAGCGCCGCCGTCTAAAAAGGTGATGTGTATTTGAGTAAAACAGCTTGCGTCATGC
GGTCGCTGCGTATATGATGCGATGAGTAAATAAACAAATACGCAAGGGGAACGCATG
AAGGTTATCGCTGTACTTAACCAGAAAGGCGGGTCAGGCAAGACGACCATCGCAAC
CCATCTAGCCCGCGCCCTGCAACTCGCCGGGGCCGATGTTCTGTTAGTCGATTCCGA
TCCCCAGGGCAGTGCCCGCGATTGGGCGGCCGTGCGGGAAGATCAACCGCTAACCG
TTGTCGGCATCGACCGCCCGACGATTGACCGCGACGTGAAGGCCATCGGCCGGCGC
GACTTCGTAGTGATCGACGGAGCGCCCCAGGCGGCGGACTTGGCTGTGTCCGCGAT
CAAGGCAGCCGACTTCGTGCTGATTCCGGTGCAGCCAAGCCCTTACGACATATGGGC
CACCGCCGACCTGGTGGAGCTGGTTAAGCAGCGCATTGAGGTCACGGATGGAAGGC
TACAAGCGGCCTTTGTCGTGTCGCGGGCGATCAAAGGCACGCGCATCGGCGGTGAG
GTTGCCGAGGCGCTGGCCGGGTACGAGCTGCCCATTCTTGAGTCCCGTATCACGCAG
CGCGTGAGCTACCCAGGCACTGCCGCCGCCGGCACAACCGTTCTTGAATCAGAACC
CGAGGGCGACGCTGCCCGCGAGGTCCAGGCGCTGGCCGCTGAAATTAAATCAAAAC
TCATTTGAGTTAATGAGGTAAAGAGAAAATGAGCAAAAGCACAAACACGCTAAGTG
CCGGCCGTCCGAGCGCACGCAGCAGCAAGGCTGCAACGTTGGCCAGCCTGGCAGA
CACGCCAGCCATGAAGCGGGTCAACTTTCAGTTGCCGGCGGAGGATCACACCAAGC
TGAAGATGTACGCGGTACGCCAAGGCAAGACCATTACCGAGCTGCTATCTGAATACA
TCGCGCAGCTACCAGAGTAAATGAGCAAATGAATAAATGAGTAGATGAATTTTAGCG
GCTAAAGGAGGCGGCATGGAAAATCAAGAACAACCAGGCACCGACGCCGTGGAAT
GCCCCATGTGTGGAGGAACGGGCGGTTGGCCAGGCGTAAGCGGCTGGGTTGTCTGC
CGGCCCTGCAATGGCACTGGAACCCCCAAGCCCGAGGAATCGGCGTGACGGTCGCA
AACCATCCGGCCCGGTACAAATCGGCGCGGCGCTGGGTGATGACCTGGTGGAGAAG
TTGAAGGCCGCGCAGGCCGCCCAGCGGCAACGCATCGAGGCAGAAGCACGCCCCG
GTGAATCGTGGCAAGCGGCCGCTGATCGAATCCGCAAAGAATCCCGGCAACCGCCG
GCAGCCGGTGCGCCGTCGATTAGGAAGCCGCCCAAGGGCGACGAGCAACCAGATTT
TTTCGTTCCGATGCTCTATGACGTGGGCACCCGCGATAGTCGCAGCATCATGGACGT
GGCCGTTTTCCGTCTGTCGAAGCGTGACCGACGAGCTGGCGAGGTGATCCGCTACG
AGCTTCCAGACGGGCACGTAGAGGTTTCCGCAGGGCCGGCCGGCATGGCCAGTGTG
TGGGATTACGACCTGGTACTGATGGCGGTTTCCCATCTAACCGAATCCATGAACCGA
TACCGGGAAGGGAAGGGAGACAAGCCCGGCCGCGTGTTCCGTCCACACGTTGCGG
ACGTACTCAAGTTCTGCCGGCGAGCCGATGGCGGAAAGCAGAAAGACGACCTGGT

AGAAACCTGCATTCGGTTAAACACCACGCACGTTGCCATGCAGCGTACGAAGAAGG
CCAAGAACGGCCGCCTGGTGACGGTATCCGAGGGTGAAGCCTTGATTAGCCGCTAC
AAGATCGTAAAGAGCGAAACCGGGCGGCCGGAGTACATCGAGATCGAGCTAGCTGA
TTGGATGTACCGCGAGATCACAGAAGGCAAGAACCCGGACGTGCTGACGGTTCACC
CCGATTACTTTTTGATCGATCCCGGCATCGGCCGTTTTCTCTACCGCCTGGCACGCCG
CGCCGCAGGCAAGGCAGAAGCCAGATGGTTGTTCAAGACGATCTACGAACGCAGT
GGCAGCGCCGGAGAGTTCAAGAAGTTCTGTTTCACCGTGCGCAAGCTGATCGGGTC
AAATGACCTGCCGGAGTACGATTTGAAGGAGGAGGCGGGGCAGGCTGGCCCGATCC
TAGTCATGCGCTACCGCAACCTGATCGAGGGCGAAGCATCCGCCGGTTCCTAATGTA
CGGAGCAGATGCTAGGGCAAATTGCCCTAGCAGGGGAAAAAGGTCGAAAAGGTCT
CTTTCCTGTGGATAGCACGTACATTGGGAACCCAAAGCCGTACATTGGGAACCGGA
ACCCGTACATTGGGAACCCAAAGCCGTACATTGGGAACCGGTCACACATGTAAGTG
ACTGATATAAAAGAGAAAAAAGGCGATTTTTCCGCCTAAAACTCTTTAAAACTTATT
AAAACTCTTAAAACCCGCCTGGCCTGTGCATAACTGTCTGGCCAGCGCACAGCCGA
AGAGCTGCAAAAAGCGCCTACCCTTCGGTCGCTGCGCTCCCTACGCCCCGCCGCTT
CGCGTCGGCCTATCGCGGCCGCTGGCCGCTCAAAAATGGCTGGCCTACGGCCAGGC
AATCTACCAGGGCGCGGACAAGCCGCGCCGTCGCCACTCGACCGCCGGCGCCCACA
TCAAGGCACCCTGCCTCGCGCGTTTCGGTGATGACGGTGAAAACCTCTGACACATG
CAGCTCCCGGAGACGGTCACAGCTTGTCTGTAAGCGGATGCCGGGAGCAGACAAG
CCCGTCAGGGCGCGTCAGCGGGTGTTGGCGGGTGTCGGGGCGCAGCCATGACCCA
GTCACGTAGCGATAGCGGAGTGTATACTGGCTTAACTATGCGGCATCAGAGCAGATT
GTACTGAGAGTGCACCATATGCGGTGTGAAATACCGCACAGATGCGTAAGGAGAAA
ATACCGCATCAGGCGCTCTTCCGCTTCCTCGCTCACTGACTCGCTGCGCTCGGTCGT
TCGGCTGCGGCGAGCGGTATCAGCTCACTCAAAGGCGGTAATACGGTTATCCACAGA
ATCAGGGGATAACGCAGGAAAGAACATGTGAGCAAAAGGCCAGCAAAAGGCCAGG
AACCGTAAAAAGGCCGCGTTGCTGGCGTTTTTCCATAGGCTCCGCCCCCCTGACGA
GCATCACAAAAATCGACGCTCAAGTCAGAGGTGGCGAAACCCGACAGGACTATAAA
GATACCAGGCGTTTCCCCCTGGAAGCTCCCTCGTGCGCTCTCCTGTTCCGACCCTGC
CGCTTACCGGATACCTGTCCGCCTTTCTCCCTTCGGGAAGCGTGGCGCTTTCTCATAG
CTCACGCTGTAGGTATCTCAGTTCGGTGTAGGTCGTTCGCTCCAAGCTGGGCTGTGT
GCACGAACCCCCCGTTCAGCCCGACCGCTGCGCCTTATCCGGTAACTATCGTCTTGA
GTCCAACCCGGTAAGACACGACTTATCGCCACTGGCAGCAGCCACTGGTAACAGGA
TTAGCAGAGCGAGGTATGTAGGCGGTGCTACAGAGTTCTTGAAGTGGTGGCCTAAC
TACGGCTACACTAGAAGGACAGTATTTGGTATCTGCGCTCTGCTGAAGCCAGTTACC
TTCGGAAAAAGAGTTGGTAGCTCTTGATCCGGCAAACAAACCACCGCTGGTAGCGG
TGGTTTTTTTGTTTGCAAGCAGCAGATTACGCGCAGAAAAAAAGGATCTCAAGAAG
ATCCTTTGATCTTTTCTACGGGGTCTGACGCTCAGTGGAACGAAAACTCACGTTAAG
GGATTTTGGTCATGCATTCTAGGTACTAAAACAATTCATCCAGTAAAATATAATATTTT
ATTTTCTCCCAATCAGGCTTGATCCCCAGTAAGTCAAAAAATAGCTCGACATACTGTT
CTTCCCCGATATCCTCCCTGATCGACCGGACGCAGAAGGCAATGTCATACCACTTGT
CCGCCCTGCCGCTTCTCCCAAGATCAATAAAGCCACTTACTTTGCCATCTTTCACAA
AGATGTTGCTGTCTCCCAGGTCGCCGTGGGAAAAGACAAGTTCCTCTTCGGGCTTTT
CCGTCTTTAAAAAATCATACAGCTCGCGCGGATCTTTAAATGGAGTGTCTTCTTCCCA
GTTTTCGCAATCCACATCGGCCAGATCGTTATTCAGTAAGTAATCCAATTCGGCTAAG
CGGCTGTCTAAGCTATTCGTATAGGGACAATCCGATATGTCGATGGAGTGAAAGAGC
CTGATGCACTCCGCATACAGCTCGATAATCTTTTCAGGGCTTTGTTCATCTTCATACTC
TTCCGAGCAAAGGACGCCATCGGCCTCACTCATGAGCAGATTGCTCCAGCCATCATG
CCGTTCAAAGTGCAGGACCTTTGGAACAGGCAGCTTTCCTTCCAGCCATAGCATCAT
GTCCTTTTCCCGTTCCACATCATAGGTGGTCCCTTTATACCGGCTGTCCGTCATTTTTA
AATATAGGTTTTCATTTTCTCCCACCAGCTTATATACCTTAGCAGGAGACATTCCTTCC
GTATCTTTTACGCAGCGGTATTTTTCGATCAGTTTTTTCAATTCCGGTGATATTCTCAT
TTTAGCCATTTATTATTTCCTTCCTCTTTTCTACAGTATTTAAAGATACCCCAAGAAGC
TAATTATAACAAGACGAACTCCAATTCACTGTTCCTTGCATTCTAAAACCTTAAATAC
CAGAAAACAGCTTTTTCAAAGTTGTTTTCAAAGTTGGCGTATAACATAGTATCGACG
GAGCCGATTTTGAAACCGCGGTGATCACAGGCAGCAACGCTCTGTCATCGTTACAAT
CAACATGCTACCCTCCGCGAGATCATCCGTGTTTCAAACCCGGCAGCTTAGTTGCCG
TTCTTCCGAATAGCATCGGTAACATGAGCAAAGTCTGCCGCCTTACAACGGCTCTCC

CGCTGACGCCGTCCCGGACTGATGGGCTGCCTGTATCGAGTGGTGATTTTGTGCCGA
GCTGCCGGTCGGGGAGCTGTTGGCTGGCTGGTGGCAGGATATATTGTGGTGTAAACA
AATTGACGCTTAGACAACTTAATAACACATTGCGGACGTTTTTAATGTACTGAATTAA
CGCCGAATTAATTCCCTAGTGTCTTTTCGCGCGGCGGCTGCACATCAGCAAACTGTG
GCCGAATGATGGGTGTTTCTGCGTGCGTGGCTAGGCATCGTCGCCCCAGCCGAGTCG
CTGCTCGCGTGTAGCCTGTGGTGTGCTGCGTCTTGGCGACGAGCGCGGAGCGACGA
CCGAGGCATGCTATTGCCAACGTCGGTGACCGCGAGCCCATCTCTGATCAGAGCCCA
GCAGCTTTGGCGGAATATTACCGCTCAATTGGACTCGTGGGCTCTAAATCGCAGCGT
TCGCTCGATAATAGATGAGGAGGACCTGGGGGCGGGACGGGTGGATCTGGTCTGAG
GGCAAGTTGGGGTAAACGGGGCCACAACCGAGCGGCTGGTTAGTTGCATATGTCTG
CTGTGCTCCGATTAATGCATAATTCGCCCGAAGATTTTCCAGCTATGACCTGAGATAG
CTCAACCCTTGCAACATCGCAGCTGGTATTCGCGAACCCGCCACACGCCACTCATTC
ATCACAAAATTGATCTAGCGGACTTGCTTCACAACAGAGCGCTCATAATTTCATAATG
CCTGAACTCACCGCGACGTCTGTCGAGAAGTTTCTGATCGAAAAGTTCGACAGCGT
CTCCGACCTGATGCAGCTCTCGGAGGGCGAAGAATCTCGTGCTTTCAGCTTCGATGT
AGGAGGGCGTGGATATGTCCTGCGGGTAAATAGCTGCGCCGATGGTTTCTACAAAGA
TCGTTATGTTTATCGGCACTTTGCATCGGCCGCGCTCCCGATTCCGGAAGTGCTTGAC
ATTGGGGAATTCAGCGAGAGCCTGACCTATTGCATCTCCCGCCGTGCACAGGGTGTC
ACGTTGCAAGACCTGCCTGAAACCGAACTGCCCGCTGTTCTGCAGCCGGTCGCGGA
GGCCATGGATGCGATCGCTGCGGCCGATCTTAGCCAGACGAGCGGGTTCGGCCCATT
CGGACCGCAAGGAATCGGTCAATACACTACATGGCGTGATTTCATATGCGCGATTGC
TGATCCCCATGTGTATCACTGGCAAACTGTGATGGACGACACCGTCAGTGCGTCCGT
CGCGCAGGCTCTCGATGAGCTGATGCTTTGGGCCGAGGACTGCCCCGAAGTCCGGC
ACCTCGTGCACGCGGATTTCGGCTCCAACAATGTCCTGACGGACAATGGCCGCATAA
CAGCGGTCATTGACTGGAGCGAGGCGATGTTCGGGGATTCCCAATACGAGGTCGCC
AACATCTTCTTCTGGAGGCCGTGGTTGGCTTGTATGGAGCAGCAGACGCGCTACTTC
GAGCGGAGGCATCCGGAGCTTGCAGGATCGCCGCGGCTCCGGGCGTATATGCTCCG
CATTGGTCTTGACCAACTCTATCAGAGCTTGGTTGACGGCAATTTCGATGATGCAGC
TTGGGCGCAGGGTCGATGCGACGCAATCGTCCGATCCGGAGCCGGGACTGTCGGGC
GTACACAAATCGCCCGCAGAAGCGCGGCCGTCTGGACCGATGGCTGTGTAGAAGTA
CTCGCCGATAGTGGAAACCGACGCCCCAGCACTCGTCCGAGGGCAAAGGAATAGTG
GCACCACTTCGTCTTCATTTGTTTTTTTTGCATATACTTGGCTTGCTTTGATTATGGTT
ATTTTGGGCTATGCTGTTTTTCTTTCCCTTACCTTTTACGATGTCGTATGAGTGAACTT
GCTTGGAATGGATTTATCTTTTTTTCTTTTTTTCTTTTCTTTTTTTTTTGGTTGCCTCGT
CTGCTTCTTCATACCCTCTTCGCCTGAAGATGAGAAAAAGTTGTTGCCAAGCGTGGA
GCACTGGAAGGAAAAGCATCGTGATGCGCTTGACAGATGAAATGAGATGAACGAGA
TTGAAAGAGAACATGATTTTCAACTTTGAGATCCAACGCCCATTCGCATCGTCATTG
TTGGCTCGGCCATTTGTTCAGTAGAAACTGTTGTATAATCCGAGTTCATGATGATATAT
CAATAATCTAGGCCGTGAGTCAAGCGGCTGTTTGTTCGACCCACCCATTGCGCGGAC
TTAACGGCCCGTAGACGTCCCTGATAACCTTTTTGAGGATCC

2. G418 expression cassette

Red: Up flanks’ region

Orange: G418 expression cassette

Blue: Down flanks’ region

ATGGCGTCTCATCCGACTCTCAAGGCTACTTTTGCATCGAGAGCTGCCACAGCCACC
CACCCTCTCAATGCCTATCTCTTCAAGTTGATGGATCTCAAAGCCTCAAATCTCTGCC
TCAGCGCCGATGTCGCAACCGCCCACGAACTTCTCTACTTTGCCGACAGGATCGGTC
CCGCCATTGTTGTCCTCAAGACTCATCATGACATGGTCTCGGGATGGGACTTTCACC
CCCAGCACGGCACTGGCGCAAAGCTCGCTGCTCTGGCCCGGCGCCATGGCTTCCTC
ATCTTCGAAGACCGCTGTCTTTTCGCGCGGCGGCTGCACATCAGCAAACTGTGGCC
GAATGATGGGTGTTTCTGCGTGCGTGGCTAGGCATCGTCGCCCCAGCCGAGTCGCTG
CTCGCGTGTAGCCTGTGGTGTGCTGCGTCTTGGCGACGAGCGCGGAGCGACGACCG
AGGCATGCTATTGCCAACGTCGGTGACCGCGAGCCCATCTCTGATCAGAGCCCAGC
AGCTTTGGCGGAATATTACCGCTCAATTGGACTCGTGGGCTCTAAATCGCAGCGTTC
GCTCGATAATAGATGAGGAGGACCTGGGGGCGGGACGGGTGGATCTGGTCTGAGGG
CAAGTTGGGGTAAACGGGGCCACAACCGAGCGGCTGGTTAGTTGCATATGTCTGCT
GTGCTCCGATTAATGCATAATTCGCCCGAAGATTTTCCAGCTATGACCTGAGATAGCT

CAACCCTTGCAACATCGCAGCTGGTATTCGCGAACCCGCCACACGCCACTCATTCAT
CACAAAATTGATCTAGCGGACTTGCTTCACAACAGAGCGCTCATAATTTCATAATGG
GCAAGGAGAAGACCCACGTCAGCCGCCCCCGCCTCAACAGCAACATGGACGCCGA
CCTCTACGGCTACAAGTGGGCCCGCGACAACGTCGGTCAGAGCGGCGCCACCATCT
ACCGCCTCTACGGCAAGCCCGACGCCCCCGAGCTCTTCCTCAAGCACGGCAAGGGC
AGCGTCGCCAACGACGTCACCGACGAGATGGTCCGCCTCAACTGGCTCACCGAGTT
CATGCCCCTCCCCACCATCAAGCACTTCATCCGCACCCCCGACGACGCCTGGCTCCT
CACCACCGCCATCCCCGGCAAGACCGCCTTCCAAGTCCTCGAGGAGTACCCCGACA
GCGGCGAGAACATCGTCGACGCCCTCGCCGTCTTCCTCCGCCGCCTCCACAGCATCC
CCGTCTGCAACTGCCCCTTCAACAGCGACCGCGTCTTCCGCCTCGCCCAAGCGCAG
AGCCGCATGAACAACGGCCTCGTCGACGCGAGCGACTTCGACGACGAGCGCAACG
GCTGGCCCGTCGAGCAAGTCTGGAAGGAGATGCACAAGCTCCTCCCCTTCAGCCCC
GACAGCGTCGTCACCCACGGCGACTTCAGCCTCGACAACCTCATCTTCGACGAGGG
CAAGCTCATCGGCTGCATCGACGTCGGCCGCGTCGGCATCGCCGACCGCTACCAAG
ACCTCGCCATCCTCTGGAACTGCCTCGGCGAGTTCAGCCCGAGCCTGCAGAAGCGC
CTCTTTCAGAAGTACGGCATCGACAACCCCGACATGAACAAGCTGCAGTTCCACCT
CATGCTCGACGAGTTCTTCTGATGGCACCACTTCGTCTTCATTTGTTTTTTTTGCATAT
ACTTGGCTTGCTTTGATTATGGTTATTTTGGGCTATGCTGTTTTTCTTTCCCTTACCTTT
TACGATGTCGTATGAGTGAACTTGCTTGGAATGGATTTATCTTTTTTTCTTTTTTTCTT
TTCTTTTTTTTTTGGTTGCCTCGTCTGCTTCTTCATACCCTCTTCGCCTGAAGATGAGA
AAAAGTTGTTGCCAAGCGTGGAGCACTGGAAGGAAAAGCATCGTGATGCGCTTGAC
AGATGAAATGAGATGAACGAGATTGAAAGAGAACATGATTTTCAACTTTGAGATCC
AACGCCCATTCGCATCGTCATTGTTGGCTCGGCCATTTGTTCAGTAGAAACTGTTGTA
TAATCCGAGTTCATGATGATATATCAATAATCTAGGCCGTGAGTCAAGCGGCTGTTTG
TTCGACCCACCCATTGCGCGGACTTAACGGCCCGTAGACGTCCCTGATAACCTTTTT
GAATGGTTCCCGGCAAAGCCTCCGTCACCTCCCTAGCCAATGCCGCCTCCAGATGGC
TTGAGCGCTACCCGTGCGAAGTGCACACTTCGGTCACCGTAGGCACTCCGACAACT
GACCAGTTTGATCCGGAGGAAGAGCAACAGGATGCCGCTCCAGATGCCCAAGAAA
CGCCAGCCGCATATCGACGAGAAGATGGCCGCAAGGGCAGTATCGTCTCCGTCACC
ACCGTCACACAAAAATACGAGTCTGCTTTGTCACCGCGCATGAGCAAGAGCATTAG
CGAAGGCGACGAGATGCTGTTC

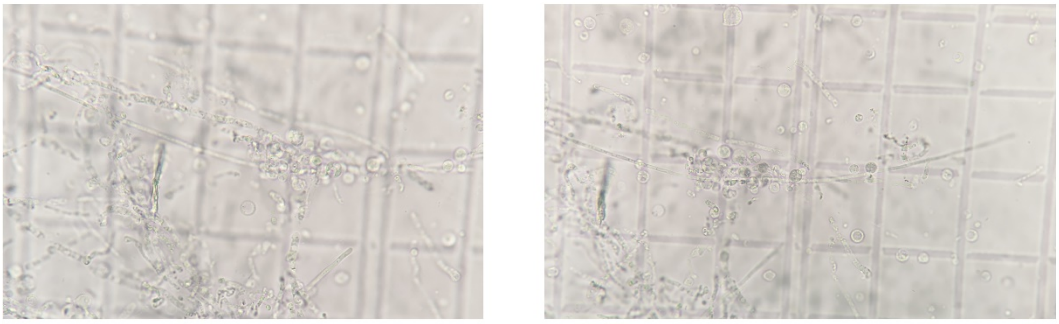

Figure S1 Protoplasts from *C. cicadae*

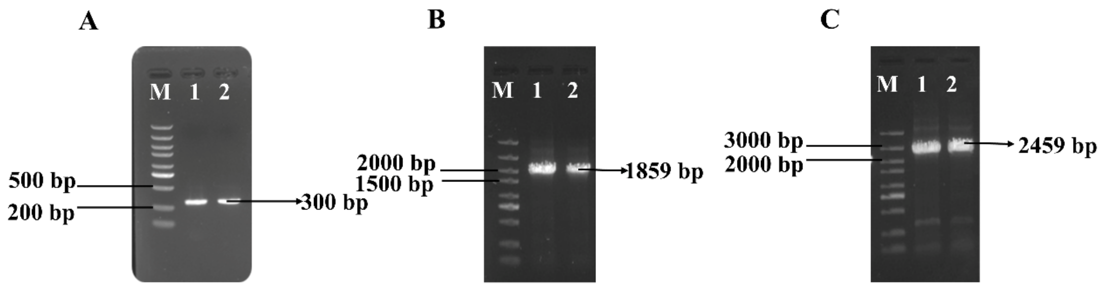

Figure S2 Construction of G418 expression cassette

(A) M:marker, 1: Up flanks' region, 2: Down flanks' region; (B) M:marker 1, 2: G418-gene; (C) 1, 2: M:marker G418 expression cassette

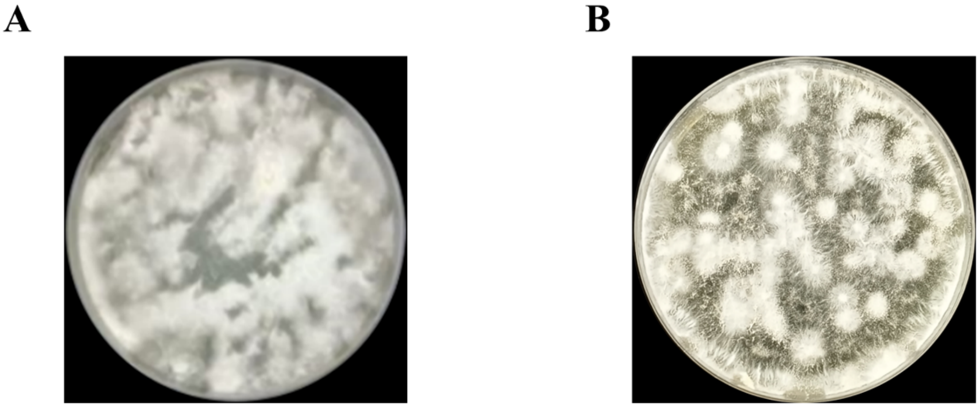

pCas9-EGFP transformants G418 expression cassette transformants

Figure S3 Colonies growth on PDA medium plates after 7 d of incubation
